# Hybrid selection for sequencing pathogen genomes from clinical samples

**DOI:** 10.1186/gb-2011-12-8-r73

**Published:** 2011-08-11

**Authors:** Alexandre Melnikov, Kevin Galinsky, Peter Rogov, Timothy Fennell, Daria Van Tyne, Carsten Russ, Rachel Daniels, Kayla G Barnes, James Bochicchio, Daouda Ndiaye, Papa D Sene, Dyann F Wirth, Chad Nusbaum, Sarah K Volkman, Bruce W Birren, Andreas Gnirke, Daniel E Neafsey

**Affiliations:** 1 Genome Sequencing and Analysis Program, Broad Institute, 7 Cambridge Center, Cambridge, MA 02142, USA; 2 Department of Immunology and Infectious Disease, Harvard School of Public Health, 665 Huntington Ave, Boston, MA 02115, USA; 3 Faculty of Medicine and Pharmacy, Cheikh Anta Diop University, BP 7325, Dakar, Senegal

## Abstract

We have adapted a solution hybrid selection protocol to enrich pathogen DNA in clinical samples dominated by human genetic material. Using mock mixtures of human and *Plasmodium falciparum *malaria parasite DNA as well as clinical samples from infected patients, we demonstrate an average of approximately 40-fold enrichment of parasite DNA after hybrid selection. This approach will enable efficient genome sequencing of pathogens from clinical samples, as well as sequencing of endosymbiotic organisms such as *Wolbachia *that live inside diverse metazoan phyla.

## Background

The falling cost of DNA sequencing means that sample quality, rather than expense, is now the blocking issue for many infectious disease genome sequencing projects. Pathogen genomes are generally very small relative to that of their human host, and are typically haploid in nature. Therefore, even a modest number of nucleated human cells present in infectious disease samples may result in the pathogen DNA representation being dwarfed relative to the host human DNA. This difference in representation poses a significant challenge to achieving adequate sequence coverage of the pathogen genome in a cost-effective manner. Separation of host and pathogen cells prior to DNA extraction can be difficult or inconvenient, particularly in field settings common to clinics in developing countries.

This barrier to the efficient sequencing of pathogen genomes comes at a time when the potential motivations and rewards for large-scale sequencing of pathogens are becoming increasingly clear. Examples abound to demonstrate how whole-genome analyses of pathogen population structure from large numbers of isolates can help to identify the source of disease outbreaks or hidden subpopulations. Whole genome sequencing of 35 *Salmonella enterica *samples was recently performed by the United States Food and Drug Administration in order to identify the source of a foodborne illness outbreak that affected approximately 300 individuals in 2009 and 2010 [1]. Whole genome sequencing of 20 isolates of pathogenic *Coccidiodies *spp. fungi identified gene flow in select genomic regions between the recently diverged *Coccidiodies immitis *and *Coccidiodies posadasii *[2]. Whole genome sequencing and comparative SNP analysis of unculturable *Mycobacterium leprae *isolates was utilized to demonstrate that a third of leprosy infections in the United States derive from armadillos [3]. So-called 'third generation' sequencing was successfully employed to identify the origin of the recent Haitian cholera outbreak strain via *de novo *sequencing of 5 isolates and comparison of those sequences to 23 previously sequenced isolates of *Vibrio cholera *[4]. In addition, the increasing use of genome-wide association studies to determine the genetic basis of important infectious disease phenotypes, such as drug resistance in malaria parasites [5,6], will require sequencing or genotyping hundreds to thousands of pathogen isolates, making a shortage of quality specimens an acute problem. All of these studies could have been performed more expediently if a culturing step were not required to eliminate DNA derived from the host or environment.

Existing methods for dealing with DNA contamination in infectious disease samples typically require significant time, money, and/or special handling of samples at the time of collection. Taking *Plasmodium falciparum *as a model case, malaria parasite samples in blood may be adapted to *in vitro *culture and sustained in a pure medium of DNA-free human red blood cells. The adaptation process, however, can take more than 6 weeks, requires considerable expertise and expense [7], and may potentially select for culturable variants. To remedy this, DNA-containing white blood cells may be depleted directly from malaria patient blood samples prior to cell lysis via differential density centrifugation or column filtration [8-10]. While depletion methodologies may reduce white cell abundance to levels useful for biochemical assays, the 100-fold disparity in genome size between human and malaria means that an even modest number of host cells can compromise a sample for genome sequencing. In addition, white blood cell depletion currently requires a significant volume of blood to be drawn from patients (approximately 5 ml), and the blood must then be stored at minus 70°C in a special medium to preserve cellular integrity. This could preclude sample collection for genome sequencing from many clinical trials due to protocol limitations or lack of equipment in the field. Furthermore, pathogens that infect or closely associate with nucleated host cells, such as *Plasmodium vivax*, *Trypanosoma cruzi*, or *Chlamydia trachomatis*, are not amenable to purification by white cell depletion. Endosymbionts such as *Wolbachia*, which influence host fertility and other traits in filarial worms, insect disease vectors, and diverse other taxa, may only be cultured in an intracellular system [11], precluding easy isolation of their genomic DNA for sequencing except by elaborate methods [12].

To address this problem we have adapted a solution hybrid selection approach originally developed for the purification of resequencing targets in the human genome [13]. In brief, biotinylated RNA probes complementary to the pathogen genome ('baits') are hybridized to pathogen DNA in solution and pulled down with magnetic streptavidin-coated beads. Host DNA is washed away and the captured pathogen DNA may then be eluted and amplified for sequencing or genotyping. We experimented with two approaches to bait design: synthetic 140-bp oligos targeting specific regions of the *P. falciparum *3D7 reference genome assembly and 'whole genome baits' (WGBs) generated from pure *P. falciparum *DNA. Using this protocol, we achieved significant enrichment of *P. falciparum *DNA, to a level that allowed us to conduct whole genome sequencing on samples that otherwise would have been prohibitively expensive to sequence.

## Results and discussion

### Hybrid selection on a mock clinical malaria sample

We performed hybrid selection with both classes of bait on a mock clinical sample consisting of 99% human DNA and 1% *Plasmodium *DNA by mass, which falls within the range of DNA ratios found in many malaria clinical samples (Table 1). Hybridization and washing steps (see Materials and methods) were carried out under standard high stringency conditions to reduce capture of host DNA. The hybrid selection protocol requires a minimum of 2 μg of input DNA (combined host and pathogen), a quantity that may not be available from many types of field samples. Therefore, we also performed hybrid selection with both bait classes on 2 μg of whole genome amplified DNA generated from 10 ng of the mock clinical sample. Quantitative PCR (qPCR) analysis indicated that whole genome amplification (WGA) does not significantly alter the fraction of malaria DNA present in the sample (post-WGA percentage *P. falciparum *DNA = 1.1 ± 0.1).

**Table 1:** Quantitative PCR enrichment measurements from 12 clinical samples

	Percentage		Parasite [DNA] (pg/μl)		
Sample	parasite DNA	WGA	Pre-hybrid selection^a^	Post-hybrid selection^a^	Fold enrichment
Th231.08 (round 1)	0.11	Yes	1.8 (0.6)	71.1 (5.6)	39.7
Th231.08 (round 2)	7.7	No	71.1 (5.6)	349.1 (74.9)	4.9
Th145.08	20	No	198.4 (17.4)	477.6 (66.7)	2.4
Th032.09	12	No	114.7 (2.9)	372.6 (59.3)	3.2
Th029.09	3	No	33.6 (0.8)	317.3 (54.7)	9.4
Th093.09	2.8	No	28.5 (1.5)	365.6 (53.4)	12.8
Th090.08	2.3	No	37.7 (1.1)	300.4 (46.9)	8.0
Th139.08	2.1	No	23.6 (0.6)	346.2 (50.7)	14.7
Th197.08	1.1	No	14.6 (0.0)	222.7 (36.1)	15.3
Th140.08	0.99	No	9.6 (0.1)	251.5 (37.4)	26.2
Th190.08	0.64	No	5.1 (0.2)	218.7 (34.0)	43.2
Th238.08	0.53	No	6.7 (0.2)	273.4 (38.1)	41.0
Th127.09	1.6	No	26.8 (0.4)	368.5 (57.1)	13.7
Th175.08	48	Yes	275.8 (7.2)	556.9 (79.4)	2.0

^a^Numbers in parentheses represent standard deviations. WGA, whole genome amplification.

Sequencing of the hybrid-selected samples revealed a significant increase in representation of *Plasmodium *DNA in every case. The synthetic baits respectively yielded an average of 41-fold and 44-fold parasite DNA enrichment for unamplified and WGA simulated clinical samples in genomic regions targeted by the baits, as measured by qPCR. Whole genome baits yielded parasite genome-wide average enrichment levels of 37-fold and 40-fold for the unamplified and WGA input samples, respectively.

**Figure 1:**
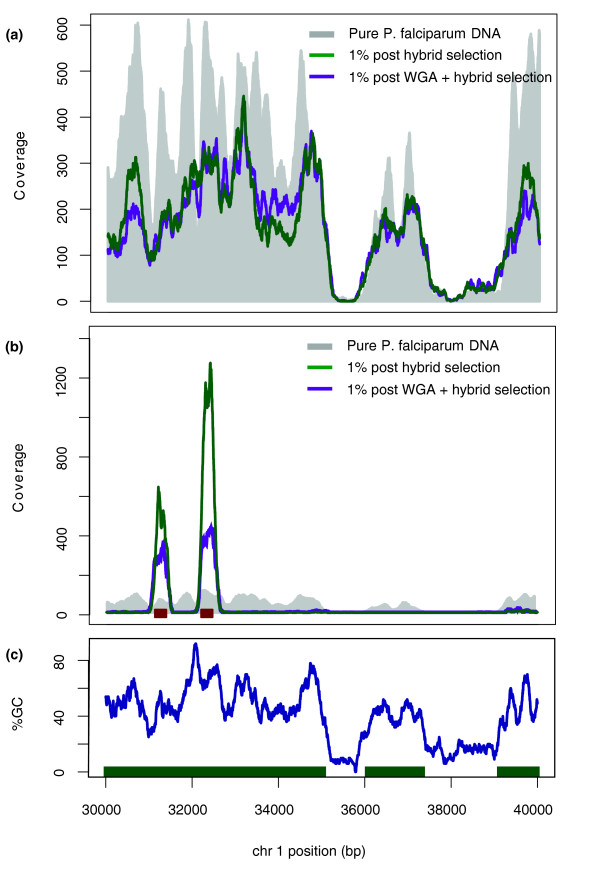
**Sequencing coverage plots from a randomly chosen region of *P. falciparum *chromosome 1**. (**a) **Unamplified (green) and WGA (purple) WGBs compared to pure *P. falciparum *(gray). **(b) **Unamplified (green) and WGA (purple) synthetic bait read coverage compared to pure *P. falciparum *(gray). Red bars indicate bait locations. **(c) **Local %GC (the percentage of nucleotides in the genome that are G or C; in 140-bp windows). Green bars indicate exons. Chr, chromosome.

Illumina sequencing coverage in the WGB hybrid-selected samples is correlated with GC content, mirroring what is observed in sequencing data from pure *P. falciparum *DNA (Figure 1a). With a genome-wide A/T composition of 81% [14], achieving uniform sequencing coverage of the *P. falciparum *genome is challenging even under ideal circumstances. Despite this challenge, we observed no reduction in coverage uniformity as a result of the hybrid selection process. WGA did not compromise mean genome-wide sequencing coverage relative to unamplified input DNA (67.5 × versus 67.1 × for a single Illumina GAIIx lane, respectively). Sequencing coverage of the samples hybrid selected using synthetic 140-bp baits was tightly localized to the genomic regions to which baits were designed (Figure 1b). Coverage levels in baited regions were significantly higher than the levels observed from comparable sequencing of pure *P. falciparum *DNA (mean coverage = 143.8 × and 92 ×, respectively; Wilcoxon rank sum test, W = 6.7E12, *P *< 2.2e-16). This indicates that hybrid selection with synthetic baits may be useful not only for reducing off-target coverage in the host genome, but also for strategically augmenting coverage levels in regions of pathogen genomes where heightened sequence coverage could be informative, such as highly polymorphic antigenic regions subject to host immune pressure.

**Figure 2:**
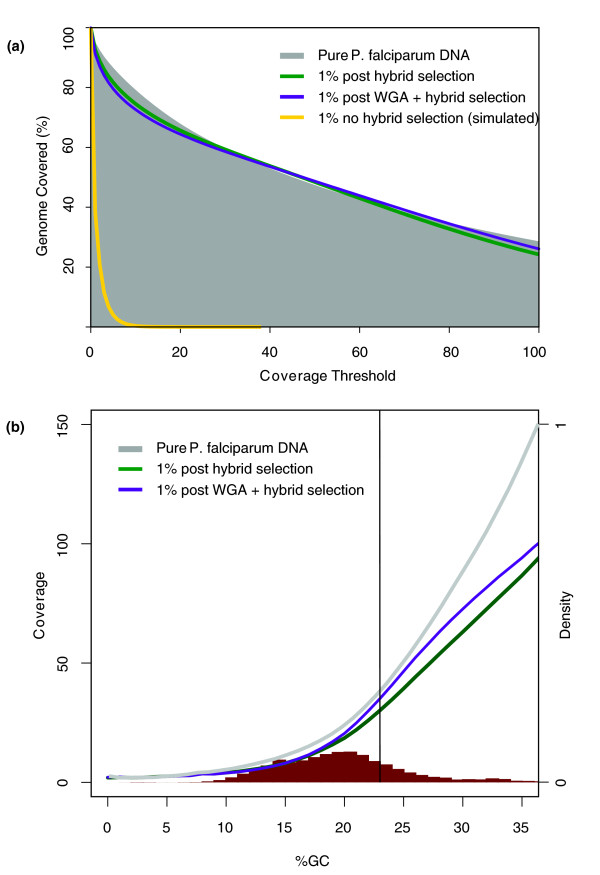
**Genome-wide sequencing coverage and composition**. (a) Coverage thresholds for unamplified (green) and WGA (purple) whole genome baits compared to pure *P. falciparum *(gray) and simulated coverage from a non-hybrid-selected mock clinical sample (yellow). **(b) **Genome-wide coverage as a function of %GC. The vertical black line represents average exonic %GC. The red histogram represents the probability density distribution of genome composition (right vertical axis). Lines depict coverage (left vertical axis) of pure *P. falciparum *DNA (gray), as well as unamplified (green) and WGA (purple) hybrid-selected samples initially containing 1% *P. falciparum *DNA. %GC, the percentage of nucleotides in the genome which are G or C.

Though effective sequencing coverage levels are reduced in the hybrid-selected mock clinical samples relative to pure *P. falciparum *DNA due to the incomplete elimination of human DNA, this reduction is small compared to the 100-fold reduction in coverage expected without hybrid selection. Genome-wide coverage is depicted in Figure 2a, which illustrates that the extent of the genome covered to various thresholds is highly similar for the pure *P. falciparum *and hybrid-selected mock clinical samples, and significantly higher than simulated coverage levels we would have predicted to have observed from sequencing an un-purified version of the sample. Genome-wide coverage levels as a function of the local %GC (the percentage of nucleotides in the genome that are G or C; %G+C) are plotted in Figure 2b for the WGB experiments. The relationship between %GC and coverage observed in whole genome shotgun sequencing data is decreased by hybrid selection due to reduced coverage in rare high %GC genomic regions (Spearman's *r_s _*for %GC versus coverage of pure malaria DNA, 0.86; versus WGB hybrid-selected DNA, 0.59; versus WGA + WGB hybrid-selected DNA, 0.64). The vertical line in Figure 2b represents the average %GC of exonic sequence (23%). Assuming a minimum threshold of 10-fold sequencing coverage is required for accurate SNP calling, 99.2% of exonic bases exhibited this coverage or greater in reads generated from the pure *P. falciparum *DNA sample. The unamplified and amplified hybrid-selected samples achieved at least 10-fold coverage for 98.3% and 98.0% of exonic bases, respectively. Given that previous pathogen population genomic analyses of outbreaks or population structure have been SNP-based [1,2,4], this indicates that sequencing data generated from hybrid-selected clinical samples could be as useful as data generated from pure pathogen DNA samples for downstream analyses. Further comparison of sequencing coverage between hybrid-selected and pure *P. falciparum *DNA indicates that local %GC and polymorphism rate do not significantly influence sequencing coverage in a hybrid-selected sample (Additional file 1).

We attempted to optimize our hybrid selection protocol by exploring two different hybridization temperatures (60°C versus 65°C) and four different 10-minute wash stringencies (0.1 × SSC, 0.25 × SSC,0.5 × SSC, and 0.75 × SSC). Eight mock clinical samples were hybridized with WGB and washed under all combinations of the above conditions. Enrichment was measured by qPCR and sequencing (one indexed Illumina GAIIx lane). We observed the best enrichment under the standard high stringency conditions used for all previously reported experiments (hybridization at 65°C and high stringency wash (0.1 × SSC). Results are presented in Table 2.

**Table 2:** Quantitative PCR enrichment measurements

Hybrid temperature	Stringency	Wash	Pre-hybrid selection [DNA] (pg/μl)	Post-hybrid selection [DNA] (pg/μl)	Fold enrichment
65°C	High	0.10 × SSC	10.0	342.9	34.3
	Med/high	0.25 × SSC	10.0	258.2	25.8
	Med/low	0.50 × SSC	10.0	227.9	22.8
	Low	0.75 × SSC	10.0	181.4	18.1
60°C	High	0.10 × SSC	10.0	288.6	28.9
	Med/high	0.25 × SSC	10.0	232.9	23.3
	Med/low	0.50 × SSC	10.0	203.5	20.4
	Low	0.75 × SSC	10.0	196.3	19.6

In summary, both bait strategies performed effectively and now offer investigators a method to sequence either targeted regions or complete genomes of pathogens in clinical samples dominated by host DNA. Pairing this hybrid selection protocol with WGA further expands the range of clinical samples now eligible for efficient pathogen genome sequencing. For example, for *Plasmodium *it should now be possible to sequence the parasite genome directly from dried blood spots on filter paper, an easily collectable and storable sample format.

### Hybrid selection on authentic clinical samples

**Figure 3:**
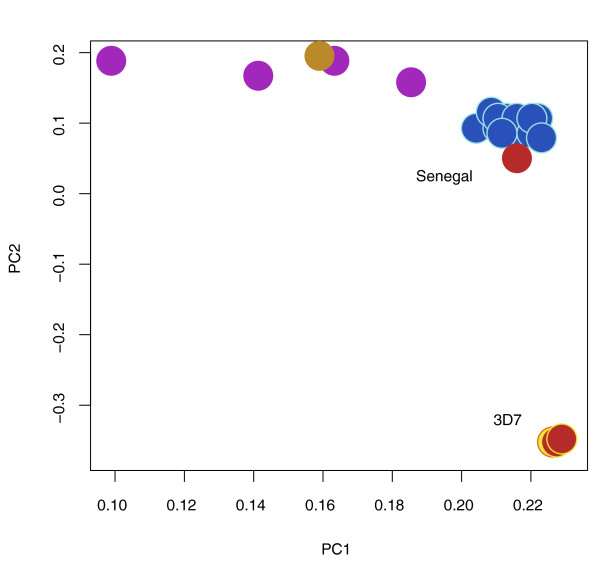
**Principal component analysis plot based on SNP calls produced from hybrid-selected and non-hybrid-selected samples**. The hybrid-selected clinical sample from Senegal (red) clusters with 12 previously sequenced Senegal samples (blue). The hybrid-selected 3D7 samples (red) cluster with the non-hybrid-selected 3D7 sample (yellow). *P. falciparum *isolates from India (purple) and Thailand (brown) are also represented. PC, principal content.

To test this application, we performed WGA and hybrid selection on DNA extracted from a clinical *P. falciparum *sample (Th231.08) collected on filter paper in Thies, Senegal in 2008 and stored at room temperature for over a year. By qPCR we estimated the *Plasmodium *DNA in the original sample to comprise approximately 0.11% of the total DNA by mass. Following WGA and hybrid selection, *Plasmodium *DNA represented 7.7% of total DNA present, an approximately 70-fold increase in parasite DNA representation. Illumina HiSeq sequencing data confirmed that at least 5.9% of mappable reads in the hybrid-selected sample corresponded to *Plasmodium*. The fraction of human reads after hybrid selection remained high due to the extreme initial ratio of host:parasite DNA, but the enrichment factor in this case was sufficient to rescue the feasibility of sequencing this sample. We evaluated the accuracy and utility of the data by calling SNPs against the *P. falciparum *reference assembly. We identified a total of 26,366 SNPs relative to the *P. falciparum *reference assembly (more than one per kilobase), close to the number of SNPs identified (33,094 to 41,123) from 11 other culture-adapted Senegalese parasite lines sequenced without hybrid selection. Further SNPs could likely be discovered by further augmenting coverage. While the depth of coverage we obtained from this experiment would not be sufficient for *de novo *genome assembly, SNP calling against a reference assembly is the end-stage analysis for most Illumina data (for example, [1-4]) and therefore a good indication of a dataset's potential utility. Principal components analysis of SNP genotypes confirms the similar genomic profile of the hybrid-selected and non-hybrid-selected Senegalese strains, as well as hybrid-selected and non-hybrid-selected 3D7 reference strain datasets generated from sequencing the mock clinical samples (Figure 3). Despite the use of WGBs generated from the 3D7 reference genome, the DNA captured from the Senegal isolate has the SNP profile of Senegal DNA, rather than 3D7 DNA, suggesting that polymorphisms do not strongly bias enrichment. In addition, the highly polymorphic regions of the isolate did not suffer a relative drop in sequencing coverage after hybrid selection. Selection of a panel of 12 other clinical malaria samples from Senegal yielded an average of 35-fold enrichment, as measured by qPCR (Table 1), with enrichment amount inversely proportional to the initial fraction of parasite DNA in the samples.

We conducted a second round of hybrid selection on the Th231.08 clinical sample to determine whether the *Plasmodium *DNA titer in the sample could be boosted above approximately 7%. The second round of hybrid selection was carried out under identical hybridization and wash conditions. qPCR analysis indicates this yielded a sample in which 47.5% of the genetic material was *Plasmodium *by mass (a 6.7-fold enrichment). This lower fold enrichment is consistent with our previous observation that fold enrichment is inversely proportional to initial parasite DNA titer, but in this case an additional round of hybrid selection yields a sample even more amenable to cost-efficient and deep sequencing.

Although sequencing has become considerably less expensive in recent years, it remains financially impractical to sequence pathogen genomes from clinical samples at scale due to the gross excess of host DNA typically present. The simplest way to compensate for host DNA contamination is to augment sequencing coverage depth. However, this strategy can be costly for all but the most lightly contaminated samples. In contrast, the cost of purification by hybrid selection using whole genome baits is approximately US$250, which is roughly equivalent to the current cost of generating 20-fold coverage of the 23 Mb *P. falciparum *genome from pure template using a fraction of an Illumina HiSeq lane. For augmented coverage to be an affordable strategy relative to hybrid selection for a target coverage level of 40 × in a genome of this size, samples must contain at least 50% pathogen DNA. This titer of parasite DNA is rarely found in clinical samples unless white cell depletion is performed prior to DNA extraction. For a more typical clinical sample containing only 1% *P. falciparum *DNA, hybrid selection resulting in 40-fold enrichment enables 40 × coverage depth for a dramatically lower total price (approximately $1,000) than deeper sequencing of the unpurified sample (approximately $40,000).

## Conclusions

The modest cost and high performance of this hybrid selection purification protocol will facilitate sequencing of archival clinical samples of malaria parasites and other pathogens previously considered unfit for sequencing by any methodology. This may enable sequencing of important samples stored on filter papers or diagnostic slides predating the spread of drug resistance or associated with historic outbreaks. This purification protocol also broadens the accessibility of sequencing for clinical samples of infectious organisms for which *in vitro *culture is possible but costly or inconvenient, such as class IV 'select agents' recognized by the Centre for Disease Control. This protocol is not limited to pathogens, and should be equally useful in sequencing commensal or symbiotic organisms closely associated with their host, such as intracellular *Wolbachia *bacteria, as was recently demonstrated by Kent et al. in their application of an array-based capture protocol [15]. The reduction in sample quality and quantity requirements permitted by hybrid selection will simplify protocol design in future large-scale clinical studies and help realize the benefits of inexpensive, massively parallel sequencing technologies for studying infectious diseases in diverse contexts.

## Materials and methods

### Samples

Mock clinical samples were generated by mixing *Homo sapiens *NA15510 DNA with a pure preparation of *P. falciparum *3D7 parasite DNA at a ratio of 99:1 (*H. sapiens*: *P. falciparum*) by mass. Samples were fluorescently quantified prior to mixing using a PicoGreen [16] assay. Authentic clinical samples were collected in 2008 from symptomatic patients at a clinic in Thies, Senegal under an approved institutional review board protocol. Samples consisted of whole blood dried and stored on a Whatman FTA card (fast technology for analysis of nucleic acids) and/or frozen whole blood stored in glycerolyte 57 solution. DNA was extracted using a DNeasy kit (Qiagen Hilden, North Rhine-Westphalia, Germany). Whole frozen blood samples yielded sufficient DNA for hybrid selection, but samples from FTA cards typically yielded less than 100 ng of DNA and required WGA. WGA was performed using the Repli-G kit (Qiagen).

### Bait design and preparation

Synthetic 140-bp oligos were obtained from Agilent and designed to capture exonic regions of the *P. falciparum *genome as defined in the 3D7 v.5.0 reference assembly. The final bait set included 24,246 oligos (3.4 Mb) with unique BLAT matches to the *P. falciparum *3D7 reference genome assembly and no homology to the human genome. Baits and locations are listed in Additional file 2. To generate synthetic single-stranded biotinylated RNA bait, *in vitro *transcription was performed with biotin-labeled UTP using the MEGAshortscript T7 kit (Ambion Austin, Texas, United States) as described previously [13].

WGB was generated at the Broad Institute. For input, 3 μg of *P. falciparum *3D7 DNA was sheared for 4 minutes on a Covaris E210 instrument set to duty cycle 5, intensity 5 and 200 cycles per burst. The mode of the resulting fragment size distribution was 250 bp. End repair, addition of a 3'-A, adaptor ligation and reaction clean-up followed the Illumina's genomic DNA sample preparation kit protocol except that adapter consisted of oligonucleotides 5'-TGTAACATCACAGCATCACCGCCATCAGTCxT-3' ('x' refers to an exonuclease I-resistant phosphorothioate linkage) and 5'-[PHOS]GACTGATGGCGCACTACGACACTACAATGT-3'. The ligation products were cleaned up (Qiagen), amplified by 8 to 12 cycles of PCR on an ABI GeneAmp 9700 thermocycler in Phusion High-Fidelity PCR master mix with HF buffer (NEB Ipswich, Massachusetts, United States) using PCR forward primer 5'-CGCTCAGCGGCCGCAGCATCACCGCCATCAGT-3' and reverse primer 5'-CGCTCAGCGGCCGCGTCGTAGTGCGCCATCAGT-3' (ABI Carlsbad, California, United States). Initial denaturation was 30 s at 98°C. Each cycle was 10 s at 98°C, 30 s at 50°C and 30 s at 68°C. PCR products were size-selected on a 4% NuSieve 3:1 agarose gel followed by QIAquick gel extraction. To add a T7 promoter, size-selected PCR products were re-amplified as above using the forward primer 5'-GGATTCTAATACGACTCACTATACGCTCAGCGGCCGCAGCATCACCGCCATCAGT-3'. Qiagen-purified PCR product was used as template for whole genome biotinylated RNA bait preparation with the MEGAshortscript T7 kit (Ambion) [13].

### Hybrid selection

Hybrid selection using either synthetic bait or WGB was carried out as described previously [13]. Hybridization was conducted at 65°C for 66 h with 2 μg of 'pond' libraries carrying standard or indexed Illumina paired-end adapter sequences and 500 ng of bait in a volume of 30 μl. After hybridization, captured DNA was pulled down using streptavidin Dynabeads (Invitrogen Carlsbad, California, United States). Beads were washed once at room temperature for 15 minutes with 0.5 ml 1 × SSC/0.1% SDS, followed by three 10-minute washes at 65°C with 0.5 ml pre-warmed 0.1 × SSC/0.1% SDS, re-suspending the beads once at each washing step. Hybrid-selected DNA was eluted with 50 μl 0.1 M NaOH. After 10 minutes at room temperature, the beads were pulled down, the supernatant transferred to a tube containing 70 μl of 1 M Tris-HCl, pH 7.5, and the neutralized DNA desalted and concentrated on a QIAquick MinElute column and eluted in 20 μl.

### Quantitative PCR enrichment measurement

Enrichment of malaria DNA in samples was assessed using a panel of malaria qPCR primers designed to conserved regions of the *P. falciparum *3D7 v.5.0 reference genome. Enrichment for each amplicon was calculated as the ratio between the amount of DNA presented pre- and post-hybrid selection, with threshold cycle (cT) counts corrected for qPCR efficiency using a standard curve for each amplicon. All qPCR reactions utilized 1 μl of template containing 1 ng of total DNA. Estimated enrichment for the samples was calculated as the mean enrichment observed across all tested amplicons. Primer sequences and locations are listed in Additional file 3. Quantification of human DNA in the clinical samples was performed prior to sequencing using the Taqman RNase P Detection Reagents kit (Applied Biosystems Carlsbad, California, United States).

### Sequencing

Each sample was sequenced at the Broad Institute using one lane of Illumina 76-bp paired-end reads. The libraries of pure *P. falciparum *DNA and hybrid-selected artificial clinical samples were each sequenced with one Illumina GAIIx lane. The hybrid-selected authentic clinical sample (Th231.08) was sequenced with one Illumina HiSeq lane. Sequence data have been deposited in the NCBI Short Read Archive under accession number [SRA029706].

### Analysis

Quality scores on Illumina reads were rescaled using the MAQ sol2sanger utility [17]. Reads were then aligned to *P. falciparum 3D7 *(PlasmoDB 5.0) using BWA [18]. Sequenced reads were sorted and the consensus sequence was determined using the SAMtools utilities [19]. %GC was calculated from 140-bp windows across the *P. falciparum *genome.

The human:*P. falciparum *DNA ratio in each sequence dataset was estimated from sequencing data by randomly sampling 50K pairs of mated reads and measuring the fractions that uniquely mapped to human versus *P. falciparum *reference genome assemblies.

Simulated sequencing read coverage for the mock clinical sample prior to hybrid selection was performed by randomly sampling 1% of the read data generated for the pure *P. falciparum *sample, under the tested assumption that read coverage scales closely with parasite DNA fraction.

Principal components analysis was performed using Eigensoft software [20] on 8,300 non-singleton SNPs with coverage of at least 10-fold in all strains and consensus quality scores of at least 30.

## Abbreviations

bp: base pair; qPCR: quantitative polymerase chain reaction; SNP: single nucleotide polymorphism; WGA: whole genome amplification; WGB: whole genome bait.

## Competing interests

The authors disclose that they are seeking to patent this application of hybrid selection and whole genome bait preparation.

## Authors' contributions

AM designed and performed the experiments, wrote the manuscript, edited the manuscript and reviewed the data. PR designed and performed the experiments. AG designed and performed the experiments, supervised the project, edited the manuscript and reviewed the data. KG performed bioinformatic analyses, edited the manuscript and reviewed the data. TF performed bioinformatic analyses. DEN performed bioinformatic analyses, supervised the project, conceived and initiated the project and wrote the manuscript. DN and PDS provided samples. DVT, KGB and RD performed DNA extractions on the samples. JB coordinated sequencing. CR and DFW supervised the project. SKV, CN and BWB supervised the project, edited the manuscript and reviewed the data. All authors have read and approved the manuscript for publication.

## Supplementary Material

Additional file 1 **Sequencing coverage comparison for 10-kb genomic windows**. Click here for file

Additional file 2 **Genomic locations of Agilent synthetic baits**. Click here for file

Additional file 3 ***P. falciparum *qPCR primers and locations (3D7 v.5.0 assembly)**. Click here for file
