# Supplementary material for: Hybrid selection for sequencing pathogen genomes from clinical samples
Source: Genome Biol. 2011 Aug 11;12(8):R73. doi: 10.1186/gb-2011-12-8-r73 (PMC3245613; doi:10.1186/gb-2011-12-8-r73)
Supplement: Additional file 1 — Sequencing coverage comparison for 10-kb genomic windows. [file gb-2011-12-8-r73-S1.PDF]

Additional File 1 - Sequencing coverage comparison for 10kb genomic windows. Points represent relative sequencing read coverage observed for10 kb genomic windows using pure *P. falciparum* DNA (x axis) vs. 1% *P. falciparum* DNA following hybrid selection (y axis) as sequencing template. Overall sequencing coverage across the genome is highly comparable between the two treatments (Pearson r^2 = 0.94), with only small number of genomic regions exhibiting disproportionately low coverage in the hybrid selected template. (**a)** Coloration according to mean G/C% **(b)** Coloration according to the frequency of SNPs segregating at high (>20%) minor allele frequency (MAF) within window from a sample of 25 sequenced Senegal strains.

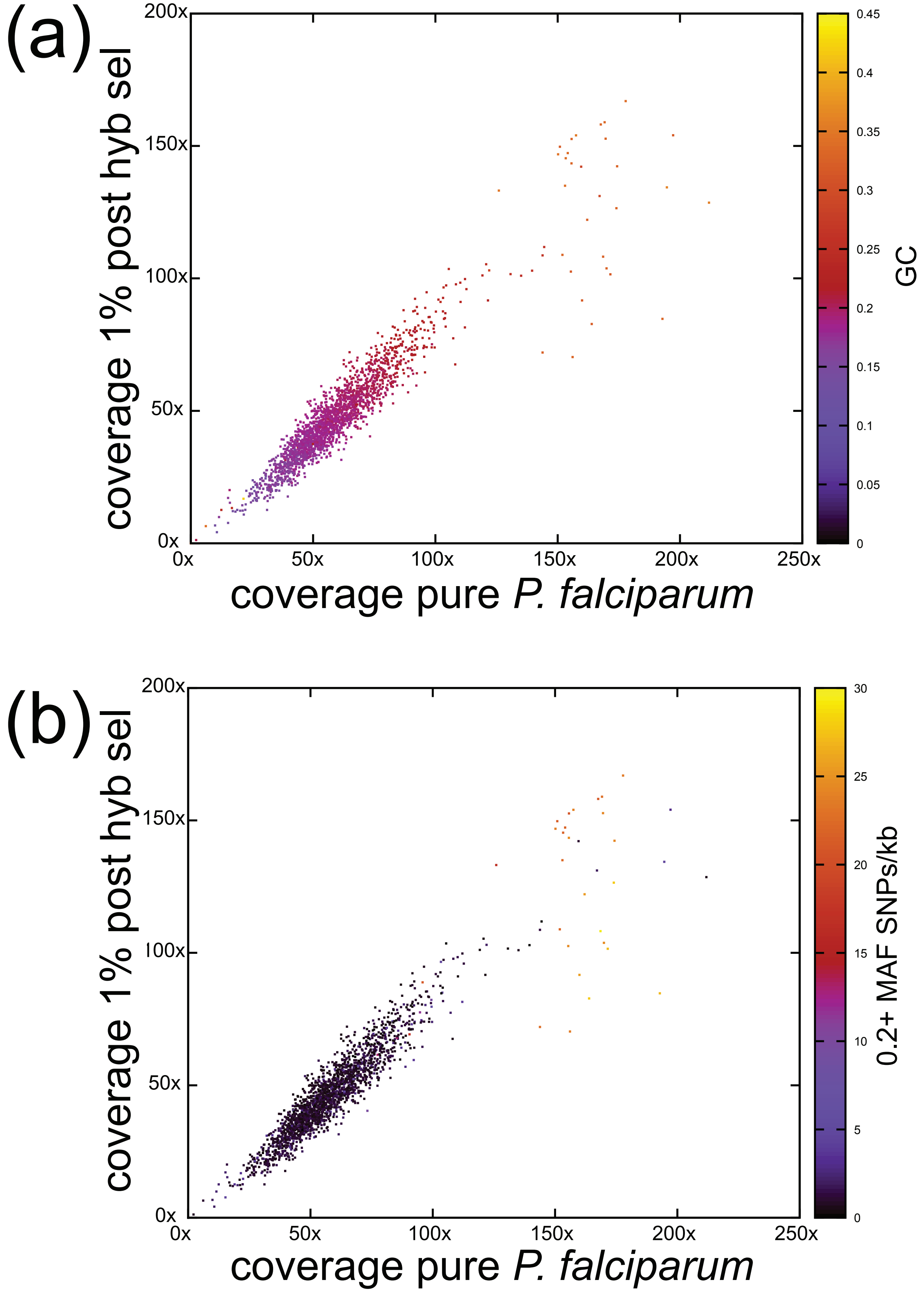

(a)
coverage 1% post hyb sel
coverage pure P. falciparum
0x
50x
100x
150x
200x
250x
GC
0
0.05
0.1
0.15
0.2
0.25
0.3
0.35
0.4
0.45
(b)
coverage 1% post hyb sel
coverage pure P. falciparum
0.2+ MAF SNPs/kb
0
5
10
15
20
25
30
