# Supplementary material for: Hybrid selection for sequencing pathogen genomes from clinical samples
Source: Genome Biol. 2011 Aug 11;12(8):R73. doi: 10.1186/gb-2011-12-8-r73 (PMC3245613; doi:10.1186/gb-2011-12-8-r73)
Supplement: Additional file 3 — P. falciparum qPCR primers and locations (3D7 v.5.0 assembly). [file gb-2011-12-8-r73-S3.DOC]

**Additional File 3.** *P. falciparum* qPCR primers and locations (3D7 v.5.0 assembly).

| Amplicon | Chr. | Start (bp) | Stop (bp) | Forward Primer (5' to 3') | Reverse Primer (5' to 3') |
| --- | --- | --- | --- | --- | --- |
| SPF1 | 2 | 629,870 | 629,921 | CGTAATTCTTCAGCAATTCTTGG | TCATATTAAATGGTGGTGGAAAG |
| SPF2 | 1 | 565,621 | 565,674 | TTGCCCATAGCTTCTCCAAC | TGCTCGAGCTTTAGTAGAAGGTG |
| SPF3 | 13 | 921,920 | 921,969 | CGGGAGCGGAATTTGATTAC | ATCATCATAATACACCGATTCGTC |
| SPF4 | 11 | 150,602 | 150,651 | CTCTGGAGCTTCACACGTTC | GGGAATGTCCAGAAAATTGG |
| SPF5 | 13 | 2,710,050 | 2,710,109 | CCTGCTGCTAGTGTTTGAGG | TTTTGATGGACAAGCAGGAG |
